# Transcriptomic resources for evolutionary studies in flat periwinkles and related species

**DOI:** 10.1038/s41597-020-0408-8

**Published:** 2020-03-03

**Authors:** João P. Marques, Graciela Sotelo, Juan Galindo, Pragya Chaube, Diana Costa, Sandra Afonso, Marina Panova, Katja Nowick, Roger Butlin, Johan Hollander, Rui Faria

**Affiliations:** 1 CIBIO, Centro de Investigação em Biodiversidade e Recursos Genéticos, InBIO Laboratório Associado, Universidade do Porto, Vairão, Portugal; 2 Departamento de Biologia, Faculdade de Ciências do Porto, Porto, Portugal; 3 Institut des Sciences de l’Évolution, Université de Montpellier, CNRS, IRD, EPHE, Montpellier, France; 4 Department of Biochemistry, Genetics and Immunology, University of Vigo, Vigo, Spain; 5 Department of Animal and Plant Sciences, University of Sheffield, Sheffield, UK; 6 Department of Marine Sciences, Centre for Marine Evolutionary Biology, University of Gothenburg, Gothenburg, Sweden; 7 Institut für Zoologie, Freie Universität Berlin, Berlin, Germany; 8 Department of Biology, Aquatic Ecology, Lund University, Lund, Sweden; 9 World Maritime University, Malmö, Sweden; 10 Interdisciplinary Centre of Marine and Environmental Research, University of Porto, Porto, Portugal

**Keywords:** RNA sequencing, Genetic variation, Genetic hybridization

## Abstract

The flat periwinkles, *Littorina fabalis* and *L. obtusata*, comprise two sister gastropod species that have an enormous potential to elucidate the mechanisms involved in ecological speciation in the marine realm. However, the molecular resources currently available for these species are still scarce. In order to circumvent this limitation, we used RNA-seq data to characterize the transcriptome of four individuals from each species sampled in different locations across the Iberian Peninsula. Four *de novo* transcriptome assemblies were generated, as well as a pseudo-reference using the *L. saxatilis* reference transcriptome as backbone. After transcripts’ annotation, variant calling resulted in the identification of 19,072 to 45,340 putatively species-diagnostic SNPs. The discriminatory power of a subset of these SNPs was validated by implementing an independent genotyping assay to characterize reference populations, resulting in an accurate classification of individuals into each species and in the identification of hybrids between the two. These data comprise valuable genomic resources for a wide range of evolutionary and conservation studies in flat periwinkles and related taxa.

## Abstract (summary)

**Table:** 

Measurement(s)	RNA • sequence_assembly • sequence feature annotation • SNP
Technology Type(s)	RNA sequencing • sequence assembly process • sequence annotation • single-nucleotide polymorphism analysis
Factor Type(s)	geographic location • species
Sample Characteristic - Organism	Littorina fabalis • Littorina obtusata
Sample Characteristic - Environment	marine biome
Sample Characteristic - Location	Iberian Peninsula

Machine-accessible metadata file describing the reported data: 10.6084/m9.figshare.11800293

## Background & Summary

**Fig. 1:**
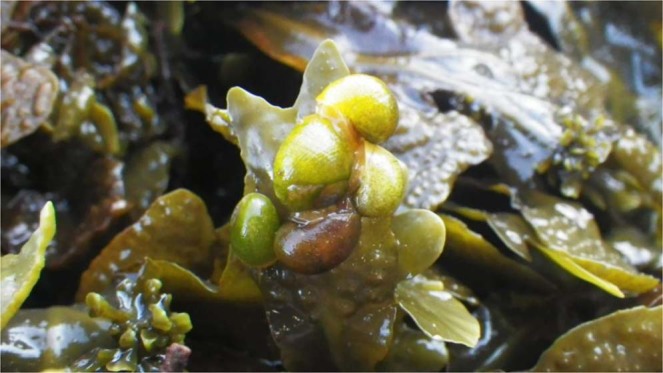
Flat periwinkles dwelling on *Fucus* spp. in the rocky intertidal of Galicia, Spain.

Marine gastropods of the genus *Littorina* are among the most interesting models for studying adaptation and speciation^1–3^. For example, the recent genomic resources made available for the rough periwinkle (*L. saxatilis*) have provided important knowledge about the genomic architecture of adaptation and of parallel ecotype evolution^4–6^. Although less studied, the flat periwinkles, *L. fabalis* and *L. obtusata*, have recently gained recognition as a model system to inform us about the late stages of ecological speciation and how different reproductive barriers accumulate and interact across the speciation continuum^7–9^. These two sister-species usually dwell on brown algae (Fig. 1), presenting a largely overlapping distribution across the European Atlantic shores^10^. In some countries (e.g. Spain and United Kingdom), their different ecological requirements result in vertical zonation, with *L. obtusata* mainly inhabiting the mid to upper intertidal, whereas *L. fabalis* is more commonly found in the lower part of the shore^11^. Despite clear morphological and ecological differences, recent studies suggest that they are not fully reproductively isolated^9,12^, with a remarkable incidence of hybrids in Cabo do Mundo (Portugal)^7,8^. However, these studies were based on a relatively small number of markers, precluding a more detailed characterization of the type of hybrids. In order to circumvent these limitations, here we use RNA-seq to increase the genomic resources available for this system. We present the first transcriptome for flat periwinkles, identify putative fixed SNP differences between the two species and demonstrate the potential of this information to detect hybrids between them. These resources will facilitate additional evolutionary studies, including the role of adaptive introgression and reinforcement in the diversification of flat periwinkles, producer-herbivore interactions, as well as the identification of genomic regions involved in key traits that diverged between rough and flat periwinkles (e.g. adaptation to live on a rocky substrate versus macroalgae, respectively). Finally, together with the first transcriptome available within the genus *Littorina* (*L. littorea)*, these data will also allow for comparative immunology studies and contribute to understanding the evolutionary dynamics of host/parasite immune conflicts^13–15^.

## Methods

**Fig. 2:**
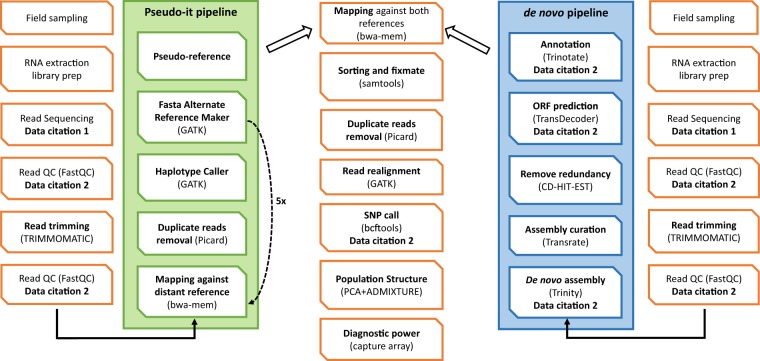
Flowchart of our pipeline. Overview of the two main approaches employed to analyse the RNA-seq data. In orange the processes common to both pipelines.

A description of the workflow of our approach is represented in a flowchart (Fig. 2).

### Sampling locations and sample processing

**Fig. 3:**
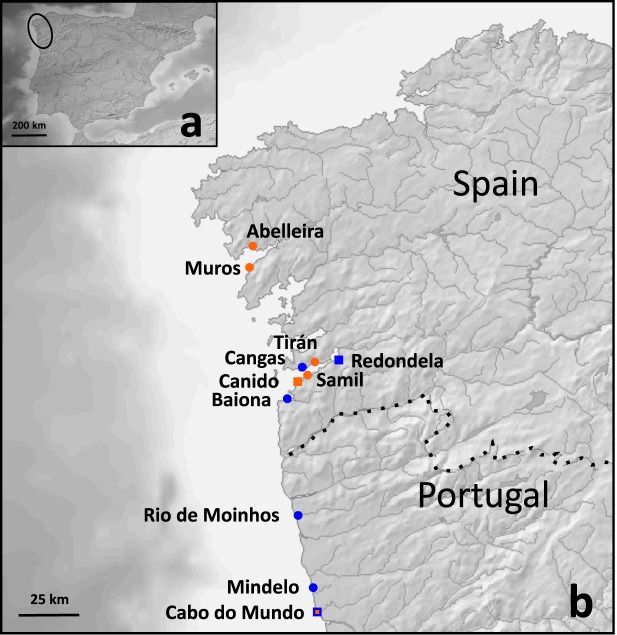
Map of the sampling locations. Samples were collected from the Northwestern Iberia (**a**). Zoom in of the sampling area is shown in (**b**). *L. obtusata* sampling sites are shown in blue, *L. fabalis* sampling sites are shown in orange, and the site where hybrids were previously described is marked with both colours. Circles represent sampling sites for transcriptome sequencing, while squares represent sampling sites for SNP genotyping/validation.

Samples for transcriptome sequencing were collected from different locations in Northwestern Iberian Peninsula, from Abelleira (Galicia, Spain) in the North to Mindelo (Portugal) in the South, during 2014. Individuals collected in each sampling site (*N* = 12 to 27) were transported alive to the laboratory (ECIMAT, University of Vigo, Spain) within 24 hours after collection. Individuals were sexed under a dissection microscope. Because the two species can be distinguished based on male genitalia morphology^10^, only males were retained. These were placed in separate aquaria (one per site and species) with running marine water and aeration during 11 to 12 days for acclimation. The maintenance of the samples in similar environmental conditions before RNA extraction was performed to ensure that differences in expression between individuals from the two species were not specifically related to their condition at collection time. The only source of nutrients during this period was the running water, to minimize sequence contamination with DNA from other organisms present in their digestive tract. At the end of the acclimation period, samples were flash frozen in liquid nitrogen and kept at ‐80 °C until further analyses. Four males per species, each from a different location, were then selected for sequencing (Table 1; Fig. 3). Forty-eight additional samples were used to test the SNPs developed in this study. These consisted of 16 *L. obtusata* from Redondela and 16 *L. fabalis* from Canido used as reference populations, as well as 16 individuals from Cabo do Mundo, where hybrids have been detected before (Table 1; Fig. 3). In order to certify that individuals were accurately assigned to one of the species, only males were analysed in the reference populations. However, in Cabo do Mundo, where admixture was previously inferred based on microsatellites, two nuclear introns and mtDNA^7–9^, both males and females were included.

**Table 1:** Sampling information. Shown is the location and respective coordinates, collection date, sample size (*N*) and species for transcriptome sequencing (locations 1 to 8), as well as for SNP genotyping (locations 9 to 11).

Location	Collection date	*N*	Species	Coordinates	
				Latitude	Longitude
1. Cangas	26.11.2014	1	*L. obtusata*	42°15′21″N	8°47′16″W
2. Baiona	21.11.2014	1	*L. obtusata*	42°07′28″N	8°50′52″W
3. Rio de Moinhos	20.11.2014	1	*L. obtusata*	41°34′00″N	8°47′50″W
4. Mindelo	09.12.2014	1	*L. obtusata*	41°18′36″N	8°44′33″W
5. Abelleira	22.11.2014	1	*L. fabalis*	42°47′53″N	9°01′30“W
6. Muros	22.11.2014	1	*L. fabalis*	42°44′34″N	8°58'53″W
7. Tirán	26.11.2014	1	*L. fabalis*	42°15′49″N	8°45'16″W
8. Samil	27.11.2014	1	*L. fabalis*	42°13′22″N	8°46′25″W
9. Redondela	03.07.2015	12	*L. obtusata*	42°17′15“N	8°37′22″W
10. Canido	18.10.2012	12	*L. fabalis*	42°11′32″N	8°48′19″W
11. Cabo do Mundo	16.11.2012; 10.09.2014; 19.03.2015	12	Admixed*	41°13′33″N	8°43′03″W

*Previously analysed by Carvalho *et al*.^7^ and Costa *et al*.^8^.

### RNA extraction

For each snail, shell and hepatopancreas were first removed (Table 2), and total RNA was extracted from all remaining tissues using the TRIzol® reagent (Invitrogen) according to manufacturer’s instructions. Residual DNA was subsequently eliminated with the TURBO DNA-free kit (Ambion) according to the manufacturer as well. RNA was purified with the RNeasy MinElute Cleanup Kit (Qiagen).

**Table 2:** Information of the samples sequenced for the transcriptome and deposited in the NCBI database, including sample ID, species and population of origin, tissues used for RNA extraction, number of raw reads obtained and NCBI BioSample ID.

NCBI Biosample ID	Library ID	Species (population)	Tissue	Raw Reads
fSAM (SAMN12385853)	G03R12	*L. fabalis* (Samil)	Whole body*	119,228,416
fABE (SAMN12385854)	G04R13	*L. fabalis* (Abelleira)	Whole body*	79,516,812
fTIR (SAMN12385855)	G10R14	*L. fabalis* (Tirán)	Whole body*	98,014,382
fMUR (SAMN12385856)	G11R15	*L. fabalis* (Muros)	Whole body*	93,801,878
oMOI (SAMN12385849)	G01R02	*L. obtusata* (Rio de Moinhos)	Whole body*	61,668,390
oCAN (SAMN12385850)	G07R05	*L. obtusata* (Cangas)	Whole body*	69,638,066
oBAI (SAMN12385851)	G08R06	*L. obtusata* (Baiona)	Whole body*	82,217,966
oMIN (SAMN12385852)	G09R07	*L. obtusata* (Mindelo)	Whole body*	132,446,602

*Includes all soft tissues except hepatopancreas.

### Library preparation and sequencing

Starting with 1 μg of RNA from each sample, library building (with individual barcodes) was performed with the TruSeq RNA Sample Preparation Kit v2 (Illumina) following the manufacturer’s protocol and aiming for insert sizes of ~200 bp. The final libraries were pooled altogether in equimolar ratios before sequencing. Sequencing was performed in two different runs (using the same pool), each consisting of 4/5 of a lane in paired-end mode (first run 2 × 100 bp and second 2 × 125 bp) on an Illumina HiSeq 1500 in CIBIO/InBIO laboratories.

### Quality control of sequence data

The quality of the raw reads^16^ was assessed with FastQC v0.11.5^17^. Subsequently, reads were clipped to remove Illumina adapters and trimmed for quality with TRIMMOMATIC v0.36^18^ with the following steps: 1. Remove the adapters; 2. Cut the initial 10 bases at the start of the reads; 3. Trimming when average quality of the nucleotides within a 4 bp window was below 15; 4. Remove bases at the start and end of the reads if quality was below 20; and 5. Discard reads shorter than 25 bp. Read quality was re-evaluated with FastQC at the end of this process^19^.

### Transcriptome assembly and annotation

In order to identify highly discriminant SNPs between *L. fabalis* and *L. obtusata*, reference transcriptomes were generated using two main approaches. The first one (Fig. 2) was based on the recently available reference transcriptome for *L. saxatilis* (deposited at DDBJ/ENA/GenBank under the accession GHPE00000000.1), a member of the sister clade of flat periwinkles closest relatives. It consisted of applying the Pseudo-it pipeline developed by Sarver *et al*.^20^ to obtain a pseudo-reference minimizing mapping biases towards any of the species. More specifically, this pipeline allows the generation of a pseudo-reference by replacing the original nucleotides in the reference (*L. saxatilis*) transcript sequences (e.g. A) by those that represent a fixed difference among the eight flat periwinkle individuals (e.g. T) or by using the IUPAC ambiguity code (e.g. R) when an alternative allele is fixed among the four *L. fabalis* (T) and the four *L. obtusata* (C) sequenced in this study. This was done through an iterative process with multiple steps, including: mapping reads to the reference using bwa mem v0.7.15^21^, removing duplicates with Picard v2.8.2 (http://broadinstitute.github.io/picard), identifying insertions and deletions following realignment and calling haplotypes with Genome Analysis Toolkit - GATK v3.7^22^, and injecting the filtered variants in the original reference making use of the FastaAlternateReferenceMaker within GATK. In line with the authors’ recommendations, five iterations were implemented to guarantee a full incorporation of species variability. Further processing (filtering, indexing, merging and sorting) was done with SAMtools v1.3.1^23^.

The second approach consisted of reconstructing species-specific *de novo* transcriptome assemblies. Four *de novo* assemblies were generated: two using the reads from the four individuals sequenced for each species (Fab4ind and Obt4ind), and the other two using only the reads from the individual with the highest coverage for each species (fSAM-G03R12 and oMIN-G09R07 for *L. fabalis* and *L. obtusata*, respectively). All these assemblies were performed using TRINITY v2.2.0^24^ with default parameters, using the cleaned paired reads. The quality and completeness of the produced assemblies was evaluated with Transrate v1.0.3^25^. This tool defines an optimal cut-off for each assembly, enabling the removal of possible chimeras and poorly supported contigs by mapping back the cleaned reads against the generated reference. Only the best-supported contigs (“good transcripts”) were retained for further analyses. In order to reduce redundancy, an extra step was performed with CD-HIT-EST v4.6.4^26^ to keep only one transcript among those with more than 95% similarity. CD-HIT-EST was used after Transrate for all *de novo* transcriptomes, except for *L. fabalis* based on multiple individuals probably due to its high complexity. Finally, TransDecoder v3.0.0^27^ was employed to predict candidate coding regions, discarding possible non-coding RNA and DNA contamination.

The final transcriptome comprises those transcripts with predicted open reading frames and with homologs in the *Littorina saxatilis* transcriptome reference plus those showing homology with PFAM common protein domains^28^. Raw and curated versions of each transcriptome are available in *Figshare*^19^. Annotation of transcripts was carried out using Conditional Reciprocal Best BLAST (crb-blast) v0.6.9^29^ against the *L. saxatilis* reference transcriptome and the Swiss-Prot database^30^ and is available in *Figshare*^19^.

### SNP calling

SNP calling was independently performed for *L. fabalis* and *L. obtusata*^16^. First, reads from all individuals were aligned to the pseudo-reference and to the respective species-specific *de novo* references with BWA-MEM v0.7.15^21^ using default parameters. Read group information was added to each sequencing lane-sample pair. SAMtools v1.3.1^23^ was used for converting the resulting alignments (SAM format) into binary files (BAM format), sorting and fixing mate-pair information. Duplicate reads were removed with Picard v1.140 using MarkDuplicates. Read realignment was performed with RealignerTargetCreator and IndelRealigner tools within GATK v3.7^22^. The pseudo-reference was chosen for downstream analyses based on mapping statistics (Table 3). However, SNPcall was also performed using the references obtained with the other assemblies^19^. Raw variant calling was carried out using bcftools mpileup and bcftools calls^31^, keeping both variant and non-variant sites and defining a maximum read coverage of 250. The resulting raw VCF files are available in *Figshare*^19^.

**Table 3:** Number and percentage of cleaned reads and mapping statistics summary.

NCBI Biosample ID	Cleaned reads	Reference									
		Pseudo-reference	(%)	Obt4ind	(%)	Fab4ind	(%)	Obt_oMIN G9R07	(%)	Fab_oSAM G3R12	(%)
fSAM (SAMN12385853)	114,099,676	34,647,272	30	29,904,860	26	31,621,272	28	25,236,448	22	35,806,196	31
fABE (SAMN12385854)	76,361,344	25,830,522	34	18,191,808	24	20,130,386	26	16,197,512	21	23,067,304	30
fTIR (SAMN12385855)	94,063,264	33,652,190	36	22,255,784	24	25,529,056	27	20,562,192	22	29,081,596	31
fMUR (SAMN12385856)	89,686,822	36,978,396	41	25,514,228	28	30,045,284	34	23,484,314	26	34,513,156	38
oMOI (SAMN12385849)	59,319,362	22,235,674	37	15,294,270	26	17,299,842	29	13,750,790	23	18,962,054	32
oCAN (SAMN12385850)	66,962,082	20,748,762	31	15,014,942	22	17,287,946	26	12,765,034	19	17,849,418	27
oBAI (SAMN12385851)	78,796,744	26,999,034	34	18,335,338	23	22,215,720	28	16,190,684	21	23,677,716	30
oMIN (SAMN12385852)	125,795,370	52,877,348	42	34,999,924	28	42,037,964	33	33,964,752	27	47,661,904	38
Total	705,084,664	253,969,198	179,511,154		164,129,506	162,151,726		230,619,344			

### Genetic variation and population structure

Genetic variability across individuals and species was first assessed by a Principal Component Analysis (PCA) using PLINK v1.90b3.45^32^, which was plotted with the ggplot2 R package^33^. This analysis was based on a subset of SNPs that were called in all eight individuals, with a minimum coverage of 20 and a minimum minor allele count of 2 (i.e. singletons excluded to avoid errors) obtained with VCFtools^34^. Furthermore, only one random biallelic SNP per transcript was retained, reducing the effect of linkage disequilibrium. Population structure analysis was performed with fastStructure v1.0^35^ based on the same SNP subset, with 10 cross-validation tests for each of three replicate runs, and considering the number of genetic clusters (*K*) between 2 and 8. The best number of clusters was inferred with the chooseK python script of fastStructure.

Putative fixed single nucleotide differences between species were identified using the python tools created by Simon Martin (https://github.com/simonhmartin/genomics_general), after filtering the raw VCF file for minimum SNP quality of 30 (Phred-scaled probability that the polymorphism exists), minimum coverage of 5 per sample and minimum number of calls of 8 (1 per individual). A more conservative set of putatively fixed SNP differences was also created by applying a more stringent filter of a minimum coverage of 20 per sample^19^.

### Gene ontology enrichment

A Gene Ontology enrichment analysis was performed to assess if the annotated subset of transcripts/genes with putatively fixed differences between *L. obtusata* and *L. fabalis* were enriched for any functional category. This analysis was performed using the more conservative set of candidate fixed SNPs with the online tool gProfiler^36^. The background panel consisted of all annotated genes with at least one SNP. Over-represented GO categories were identified using a hypergeometric test with a significance threshold of p < 0.05 after Benjamini-Hochberg correction^37^.

### Selection of informative SNPs for validation

A genotyping assay was designed with the goal of validating a subset of the identified SNPs and assessing their informativeness for species discrimination. Sequences of 101 bp with a fixed nucleotide difference in the central position were randomly selected from the list of annotated contigs/genes containing putative diagnostic SNPs (less conservative set). Sequences containing at least one polymorphism across 50 bp to each side of the target SNP were discarded to reduce the probability of missing genotypes or null alleles. Finally, to ensure that the 101 nucleotides were located in the same exon avoiding fragments that span introns, all the selected fragments were blasted against the *L. saxatilis* genome^38^ and only those with a single hit of 101 bp continuous alignment with a maximum of 1 bp difference were retained for developing the assay.

### SNP genotyping and informativeness

A final set of 42 putatively diagnostic SNPs was tested with an independent genotyping assay at the National Genomics Infrastructure (NGI), SNP&SEQ Technology Platform (www.genotyping.se) at Uppsala University, Sweden. This consisted of using multiplexed primer extension (iPLEX) and a MassARRAY analyzer^39^ from Agena Bioscience for allele detection by mass spectrometry, and subsequent conversion to a genotype using Typer (Agena Bioscience). Forty-eight samples from the Iberian Peninsula were genotyped (Fig. 3, Table 1). Hardy-Weinberg equilibrium (HWE) was estimated for each locus and reference population combination. Since there was no variability for most SNPs within the reference populations, linkage disequilibrium (LD) between locus pairs was evaluated across all populations. Both HWE and LD (genotypic disequilibrium) were evaluated through exact probability tests in Genepop v4.2^40,41^, using a Markov chain with default parameters values. A Bonferroni correction for multiple tests was applied. After removing markers in LD or not conforming to HWE, the ancestry of each individual (*L. fabalis*, *L. obtusata*, or admixed) was inferred using STRUCTURE v2.3.4^42–44^, running 10 replicates for *K* = 2, each consisting of 1,000,000 iterations after a 100,000 burn-in. Since all replicates resulted in a similar outcome, the one with the highest likelihood was plotted with DISTRUCT v1.1^45^.

## Data Records

The core dataset of this work comprises the transcriptomic data of flat periwinkles. The raw data (eight SRA experiments) were deposited in *NCBI Sequence Read Archive*, with the BioProject accession number PRJNA556984^16^ (Table 1). FASTQ files were divided by species (*L. obtusata* - Lobt and *L. fabalis* - Lfab) and biosample-specimen (oMOI, oCAN, oBAI, oMIN, fSAM, fABE, fTIR and fMUR). For each biosample, four files were submitted, corresponding to two different Illumina HiSeq lanes and one for each of the paired reads. All intermediate and final files produced during the previous steps are available on *Figshare*^19^, including: pre- and post-cleaning FASTQC quality reports (FastQC_quality_reports_raw and FastQC_quality_reports_clean, the pseudo reference transcriptome (LfabLobt_5it.pseudoref.FINAL.fa), the raw trinity assembly results (Lobt4ind_rawAssembly.fasta, Lfab4ind_rawAssembly.fasta, Lobt_oMIN_ G09R07_rawAssembly.fasta and Lfab_fSAM_G03R012_rawAssembly.fasta), curated versions of the transcriptome assemblies (Lobt4ind_curatedAssembly.fasta, Lfab4ind_curatedAssembly.fasta, Lobt_oMIN_G09R07_curatedAssembly.fasta and Lfab_fSAM_G03R012_curatedAssembly.fasta), annotated pseudoreference transcriptome based on Swiss-Prot and the *L. saxatilis* transcriptome (LfabLobt_5it.pseudoref.annotated.fa) and combined crb-blast annotation file based on Swiss-Prot and the *L. saxatilis* transcriptome (Annotation_PseudoRef_SP_and_LsaxTranscriptome.txt), transcriptomes crb-blast annotation files based on Swiss-Prot and the *L. saxatilis* transcriptome, separately (Annotation.7z), raw SNP call VCF files (SNPcall_rawPseudoRef.vcf.gz, SNPcall_rawLobt4ind.vcf, SNPcall_rawLfab4ind.vcf.gz, SNPcall_rawLob_oMIN_ G09R07.vcf.gz, SNPcall_rawLfab_fSAm_G03R012.vcf.gz), filtered VCF file (SNPcall_Biallelic_NoMissing_PseudoRef_min20.vcf), list of putatively diagnostic SNPs (FixedSNPs_PseudoRef_min5.vcf and FixedSNPs_PseudoRef_min20.vcf), and genotypes of the samples used for SNP validation analysed with Structure (SNPvalidation_genotypes_for_Structure_Analyses.xlsx). The *L. saxatilis* transcriptome and genome sequence data used as reference in this study are publically available at GenBank (GHPE00000000.1)^46^.

## Technical Validation

### RNA integrity

RNA quality was evaluated after electrophoresis on the Bioanalyser 2,100 (Agilent Technologies) with the RNA 6000 Nano Chip Kit (Agilent Technologies). RNA quantity was measured with Qubit using the RNA BR Assay Kit (Life Technologies).

### RNA-Seq data quality

A total of 736 million reads were generated (368,266,256 paired-end reads), with an average of ~92,000 reads per sample (Table 3). From these, ~705 million reads (96%) were kept after removing the adapters and trimming for quality. These post-cleaning reads passed the minimum quality standards of FastQC^17^.

### Pseudo-reference information

The pseudo-reference transcriptome is composed of 37,873 contigs (containing a total of 82,360,655 bp) with a mean contig length and N50 of 2,139 bp and 2,963 bp, respectively (Table 4). Annotation using a conditional reciprocal best blast hit approach against the Swiss-Prot database coding sequences resulted in 10,968 annotated transcripts (29%), while 91% of the transcripts had 1to1 orthologs in the *L. saxatilis* reference transcriptome.

**Table 4:** Summary statistics for the transcriptome pseudo-reference assembly.

Periwinkles Pseudo-reference	Value
Raw reads	736,532,512
Cleaned reads	705,084,664
Number of contigs	37,873
Largest (bp)	18,165
Smallest (bp)	297
N50 (bp)	2,963
Mean (bp)	2,139
Swiss-Prot annotated transcripts (%)	29

### Species-specific transcriptome assembly and annotation

The *L. fabalis* assembly based on the “cleaned” reads from 4 individuals of this species initially resulted in 396,047 contigs with a mean length of 612 bp, a N50 of 832 bp and a total size of 242,362,528 bp; whereas the *L. obtusata* transcriptome is 251,985,325 bp long, distributed across 349,459 contigs with a mean length of 721 bp and N50 of 1,133 bp (Table 5). The assemblies based on a single individual from each species resulted in lower values for all parameters when compared to those based in all four individuals: 177,022 contigs with N50 of 715 bp for *L. fabalis*; and 208,362 contigs with N50 of 1,159 bp for *L. obtusata* (Table 5).

**Table 5:** Summary statistics of the *Littorina obtusata* and *L. fabalis* transcriptome assemblies.

Samples	*L. fabalis*	*L. obtusata*	*L. fabalis*	*L. obtusata*
	fSAM_G03R12	oMIN_G09R07	4ind^#^	4ind^#^
Raw reads	119,228,416	132,446,602	390,561,488	345,971,024
Clean reads	114,099,676	125,795,370	374,211,106	330,873,558
**Raw** ***de novo*** **assembly (Trinity)**				
Number of contigs	177,022	208,362	396,047	349,459
Largest (bp)	15,009	16,581	17,410	29,198
Smallest (bp)	201	201	201	201
N50 (bp)	715	1,159	832	1,133
Mean (bp)	566	743	612	721
**Post assembly curation (TransRate)**			CD-HIT-EST*	
Number of contigs	141,456	102,412	325,814	186,239
Largest (bp)	15,009	11,334	17,410	29,198
Smallest (bp)	201	201	201	201
N50 (bp)	696	773	676	668
Mean (bp)	558	607	547	546
**Post redundancy removal (CD-HIT-EST)**			TransRate*	
Number of contigs	133,230	101,042	225,829	180,798
Largest (bp)	15,009	11,334	17,410	29,198
Smallest (bp)	201	201	201	201
N50 (bp)	660	775	799	665
Mean (bp)	540	607	612	544
**Post ORF prediction (TransDecoder)**				
Number of contigs	31,279	24,047	53,214	32,433
Largest (bp)	11,904	11,232	13,317	28,629
Smallest (bp)	255	258	255	258
N50 (bp)	801	912	1,080	861
Mean (bp)	685	740	818	707
*Littorina saxatilis* transcriptome coverage (%)	17	25	27	34

*Due to the complexity of the *L. fabalis* assembly based on multiple individuals, the order of the curation steps was reversed.

^#^Based on the data from the four individuals from each species described in Table 2.

Filtering of these contigs based on CD-HIT-EST and TransRate optimal assembly score (0.08 for *L. obtusata* four individuals, 0.19 for *L. fabalis* four individuals, 0.12 one individual *L. obtusata* and 0.09 one individual *L. fabalis*), resulted in the retention of 48.5% (*L. obtusata* one individual) to 75.3% (*L. fabalis* one individual) of the initial number of contigs. Final curated flat periwinkle assemblies based on four individuals are composed of 53,214 and 32,433 predicted open reading frame (ORF) transcripts with mean lengths of 818 bp and 707 bp, and a N50 length of 1,080 and 861, for *L. fabalis* and *L. obtusata* respectively. The single individual assemblies are composed of 31,279 (fSAM) and 24,047 (oMIN) ORF, with mean lengths of 685 bp and 740 bp, and a N50 length of 801 and 912, respectively (Table 5).

Regarding the annotation of the assemblies based on data from four individuals, a total of 38,439 transcripts in the *L. fabalis* and 23,237 in *L. obtusata* were annotated (72% in both species), of which 10,592 and 6,275 were uniquely annotated to the Swiss-Prot database, while 20,246 and 15,690 had homologs in the *L. saxatilis* reference transcriptome. The annotation of the assemblies based on a single individual resulted in 23,947 annotated transcripts for *L. fabalis* (fSAM) and 19,228 for *L. obtusata* (oMIN)^19^.

### Mapping, SNP calling, genetic differentiation and gene ontology enrichment

**Fig. 4:**
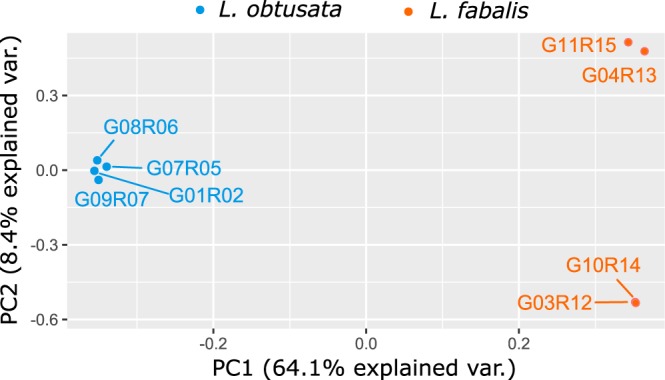
Principal Component Analysis (PCA) for the eight flat periwinkle samples sequenced for the transcriptome based on a total of 7,061 SNPs randomly sampled from independent transcripts.

**Fig. 5:**
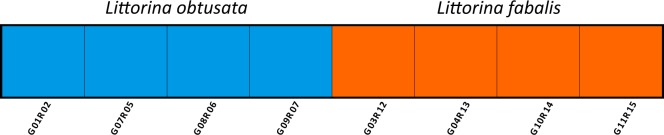
Admixture plot showing the membership of each individual sequenced for the transcriptome to the two genetic clusters. Sample codes are the same as in Table 1. No signatures of admixture were found in these individuals. The analysis was based on 7,061 random SNPs from different transcripts.

In total, 253,969,198 reads (mean = 36%) were mapped to the pseudo-reference transcriptome (Table 3). The relatively small number of mapped reads can be due to the large number of transcripts filtered out during the assembly, and also possibly due to exogenous DNA. After filtering, a total of 90,826 SNPs, present in all samples, were inferred (SNPcall_Biallelic_NoMissing_PseudoRef_min20.vcf). Principal Component Analysis was based on the filtered dataset, from which 7,061 SNPs were randomly sampled from independent transcripts. The first component of the PCA explains 64% of the total variance and separates the individuals from different species, while the second component, explains 8% of the variance and essentially separates the *L. fabalis* individuals according to geographical distance: two from Ria de Vigo (fSAM-G03R12 and fTIR-G10R14) and two from Ria de Muros (fABE-G04R13 and fMUR-G11R15) (Fig. 4). The Bayesian assignment analysis with fastStructure, with *K* = 2 as the number of clusters that maximized the marginal likelihood and better explained the structure in the data, confirms the classification of the four individuals from each species, without any evidence of admixture (Fig. 5).

Putative species-diagnostic SNPs were identified from the raw SNP call using the python tools created by Simon Martin and two different filtering criteria. In the more stringent filter (minimum coverage of 20), 19,072 SNPs were inferred as putatively species-diagnostic (i.e. alternative alleles fixed within the group of four individuals from each species) whereas in the less stringent filter (minimum coverage of 5), 45,340 were identified as putatively species-diagnostic SNPs (FixedSNPs_PseudoRef_min5.vcf and FixedSNPs_PseudoRef_min20.vcf, respectively^19^).

Among the genes with fixed differences between species no enrichment for a particular GO term was found. However, since RNA was extracted basically from the whole individual (not from individual tissues) and many of the transcripts may not be complete, this result should be interpreted with caution.

### SNP genotyping validation and informativeness

From the 42 putatively diagnostic SNPs selected for validation, two failed the quality control tests for primer design. Among the remaining SNPs, one failed during genotyping, one revealed no variability and three others revealed more than two alleles. All these SNPS were excluded resulting in a final set of 35 SNPs. All loci were genotyped for at least 91.7% of the individuals, whereas all individuals were genotyped for at least 94.3% of the loci. No conflicts were found among genotyping replicates (215), resulting in 100% reproducibility. Among these 35 loci, 26 (74.3%) SNPs are potentially diagnostic between *L. fabalis* and *L. obtusata*, i.e. fixed differences between the putatively pure populations. The non-diagnostic set consisted of two (5.7%) nearly diagnostic SNPs (one allele with a frequency ≥0.95 in one species and 0 in the other), five (14.2%) informative SNPs (one allele with a frequency ≥0.70 in one species and 0 to 0.03 in the other), and two others (5.7%) with very little or no discriminatory power between the two species defined morphologically according to male genitalia.

**Fig. 6:**
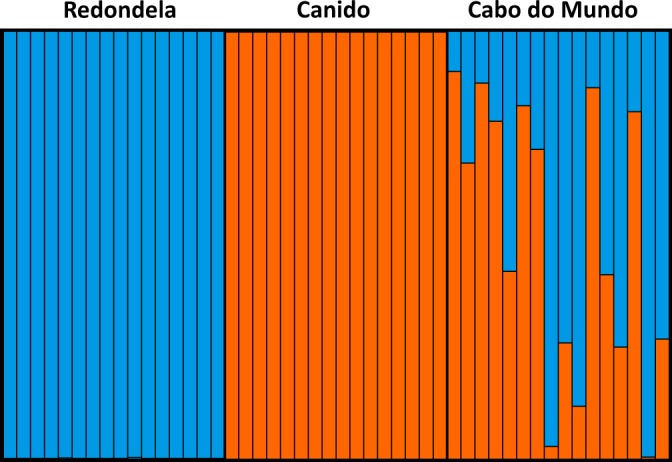
Structure plot showing the membership of each individual genotyped for a subset of putatively diagnostic SNPs to each genetic cluster. Each column represents one individual. Signatures of admixture were only found in Cabo do Mundo, whereas individuals from Redondela (*L. obtusata*) and Canido (*L. fabalis*) represent two different genetic clusters.

No significant Hardy-Weinberg deviations for any of the 35 loci were detected in the reference samples. However, LD remained significant between eight pairs of loci across the three populations. This resulted in the exclusion of six additional SNPs to avoid using non-independent information in subsequent analysis of ancestry. The STRUCTURE analysis for *K* = 2 based on these 29 SNPs confirmed the pure genetic ancestry of the reference *L. fabalis* and *L. obtusata* individuals and the occurrence of admixture in Cabo do Mundo, demonstrating the usefulness of these markers for species discrimination and detection of hybridization between flat periwinkles (Fig. 6).

## Usage Notes

The two species-specific transcriptomes and the SNPs made available in this study are the first genomic/transcriptomic resources made publicly available for flat periwinkles. This information will be useful for a wide range of studies, including evolutionarily-oriented projects concerning adaptation, parallel evolution, comparative immunology, producer-herbivore interactions, hybridization and speciation; as well as for characterization of population genetic diversity and differentiation across the species’ distribution ranges to help implementing appropriate management and conservation measures. However, it is important to emphasise that the discriminatory power of the SNPs here developed was based on individuals from the Iberian Peninsula. Although we expect that most of these SNPs will also be diagnostic in other regions, their power to discriminate between *L. fabalis* and *L. obtusata* should first be tested in other parts of the species distribution range before they are applied outside Iberia.

## Data Availability

1. FASTQC v0.11.5^17^, options: default 2. Trimmomatic v0.36^18^, options: PE ILLUMINACLIP:Adapters.fa:2:30:10 HEADCROP:10 SLIDINGWINDOW:4:15 LEADING:20 TRAILING:20 MINLEN:25 3. Pseudo-it v1^20^, options: –PE1 R1.fastq.gz –PE2 R2.fastq.gz –iupac 5 REF NAME 4. Trinity v2.2.0^24^, default 5. Transrate v1.0.3^25^, options: –left –right –reference 6. CD-HIT-EST v4.6.4^26^, options: -c 0.95 -M 0 7. TransDecoder.LongOrfs v3.0.0^27^, default 8. hmmscan v v3.1b2^47^, options: default 9. TransDecoder.Predict v3.0.0^27^, options: –retain_pfam_hits–retain_blastp_hits –single_best_only 10. crb-blast v0.6.9^29^, options: -e 1.0e-05 11. BWA v0.7.15^21^, options: mem -M -R ‘@RG\tID:\tLB:\tPL:\tSM:’ 12. Samtools v1.3.1^23^, options: sort; fixmate, index 13. Picard v1.140 (“Picard Toolkit.” 2019. Broad Institute, GitHub Repository: http://broadinstitute.github.io/picard/), options: MarkDuplicates REMOVE_DUPLICATES = true ASSUME_SORTED = true 14. GATK v3.7^22^, options: RealignerTargetCreator; IndelRealigner 15. BCFtools v1.6^31^, options: mpileup -f REF.fa -Ou -a DP -b list|bcftools call -m -f GQ,GP 16. Plink v1.90b3.45^32^, options: –bp-space 10000 –pca 17. VCFtools v. 0.1.14^34^, vcftools –gzvcf target.vcf.gz–max-missing 1.0 –min-alleles 2 –max-alleles 2 –mac 2 –remove-indels–recode–stdout|bgzip -c > Filtered.biallelic.vcf.gz 18. fastStructure v1.0^35^, options: –full –cv = 10 19. Simon Martin’s scripts (Simon Martin, GitHub Repository: https://github.com/simonhmartin/genomics_general accessed on July 2018), options: parseVCF.py –skipIndels –minQual 30 –gtf flag = DP min = 20 (or 5); filterGenotypes.py –fixedDiffs–minCalls 8 20. Genepop v4.2^40,41^, options: 1. Hardy Weinberg Exact Tests and 2. Linkage disequilibrium, default 21. STRUCTURE v2.3.4^42–44^, options: burnin = 100,000, numreps = 1,000,000, usepopinfo = 0; inferalpha = 1; maxpops = 2.
